# Besides Huntington's disease, does brain-type creatine kinase play a role in other forms of hearing impairment resulting from a common pathological cause?

**DOI:** 10.18632/aging.100338

**Published:** 2011-06-15

**Authors:** Yow-Sien Lin, Chih-Hung Wang, Yijuang Chern

**Affiliations:** ^1^ Molecular Medicine Program, National Yang-Ming University, Taipei, Taiwan;; ^2^ Institute of Neuroscience, National Yang Ming University; Taipei, Taiwan;; ^3^ Division of Neuroscience, Institute of Biomedical Sciences, Academia Sinica, Taipei, Taiwan;; ^4^ Department of Otolaryngology-Head and Neck Surgery, Tri-Service General Hospital, National Defense Medical Center, Taipei, Taiwan;; ^5^ Institute of Microbiology and Immunology, National Defense Medical Center, Taipei, Taiwan;; ^6^ Institute of Undersea and Hyperbaric Medicine, National Defense Medical Center, Taipei, Taiwan

**Keywords:** brain-type creatine kinase, Huntingtin, Huntington's disease, creatine, hearing loss, cochlea

## Abstract

Hearing impairment following cochlear damage due to noise trauma, ototoxicity caused by aminoglycoside antibiotics, or age-related cochlear degeneration was linked to a common pathogenesis involving the formation of reactive oxygen species (ROS). Cochleae are more vulnerable to oxidative stress than other organs because of the high metabolic demands of their mechanosensory hair cells in response to sound stimulation. We recently showed that patients and mice with Huntington's disease (HD) have hearing impairment and that the dysregulated phosphocreatine (PCr)-creatine kinase (CK) system may account for this auditory dysfunction. Given the importance of noninvasive biomarkers and the easy access of hearing tests, the symptom of hearing loss in HD patients may serve as a useful clinical indicator of disease onset and progression of HD. We also showed that dietary creatine supplementation rescued the impaired PCr-CK system and improved the expression of cochlear brain-type creatine kinase (CKB) in HD mice, thereby restoring their hearing. Because creatine is an antioxidant, we postulated that creatine might enhance expression of CKB by reducing oxidative stress. In addition to HD-related hearing impairment, inferior CKB expression and/or an impaired PCr-CK system may also play an important role in other hearing impairments caused by elevated levels of ROS. Most importantly, dietary supplements may be beneficial to patients with these hearing deficiencies.

## INTRODUCTION

**Figure 1:**
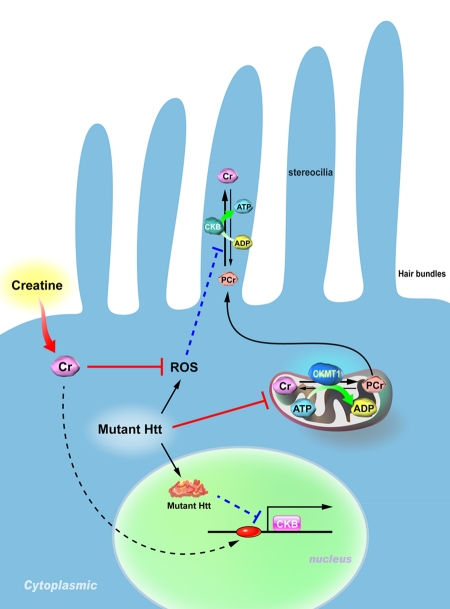
Proposed model for the pathogenesis of hearing impairment in Huntington's disease (HD). Mitochondrial creatine kinase (CKMT1) phosphorylates creatine (Cr) and converts it to phosphocreatine (PCr), while brain-type creatine kinase (CKB) regenerates ATP from PCr. Because the stereocilia contain no mitochondria, the PCr-CK system plays a critical role in hair bundles of hair cells. Expression of mutant Huntingtin (Htt) in hair cells impairs the functioning of mitochondria, suppresses the expression of CKB, and elevates levels of reactive oxygen species (ROS). Creatine supplementation in HD mice ameliorates the reduced expression of CKB via an unidentified pathway, and subsequently improves the hearing impairment in HD mice.

Huntington's disease (HD) is an autosomal dominant neurodegenerative disorder with onset usually in middle age. Clinical features of HD include uncontrollable motor movements, cognitive impairment, and psychiatric symptoms [1]. Although the causative gene (Huntingtin, *HTT*) of HD is ubiquitously expressed, the polyglutamine (polyQ)-expanded mutant Huntingtin protein (Htt) forms nuclear and neutrophil aggregates and preferentially affects the striatum and cerebral cortex. In addition to altered functions in the central nervous system, the expression of mutant Htt was also found in peripheral tissues [2-4], and was directly linked to local tissue defects [5, 6]. We recently reported that patients and mice with HD have hearing impairment [7], for which an association between dysregulated brain-type creatine kinase (CKB) and impaired hearing in HD mice was demonstrated. Expression levels of CKB in the cochlea of two different HD mice models (R6/2 and Hdh^(CAG)150^) were significantly lower than that of WT mice, suggesting that the impairment of CKB in the cochlea is likely an authentic defect of HD. Interestingly, dietary creatine supplements to HD mice not only rescued the expression of cochlear CKB but also restored the hearing of HD mice (Figure 1) [7]. It would be of great interest in the future to evaluate whether hearing loss of HD patients can be treated by dietary creatine supplements.

Our findings indicate that the impairment of CKB may account for the cochlear energy deficiency which is likely a primary cause of the observed hearing loss in HD mice [7]. Because the hearing system involves high-energy-demanding metabolic processes, CKB is likely to play an important role in maintaining normal hearing, as well as in pathological hearing impairments caused by energy deficiencies in the cochlea. In this research perspective, we suggest that hearing loss may serve as a biomarker to monitor the progression of HD and discuss the potential roles of CKB and the phosphocreatine (PCr)-creatine kinase (CK) system in neurodegenerative disorders associated with energy deficits.

### CKB and HD

It is well known that CK regulates ATP regeneration and *energy homeostasis* by catalyzing the reversible transfer of high-energy phosphate from phosphocreatine to ADP [8-11]. Tissues such as the brain, skeletal and cardiac muscles, retinas, and spermatozoa express large amounts of CK to produce adequate energy stores for dynamic energy requirements [12-17]. It is important to note that the level of CKB was lower in the cochlea of HD mice, where aggregations of mutant Htt (Nlls) were also present [7]. The effect of Nlls on the structure and function of the cochlea and the interplay between Nlls and CKB levels are currently unknown and are worthy of further evaluation in the future. Most importantly, we found that hearing impairment was closely associated with motor deficits, a major symptom of HD patients [7]. To date, reliable biomarkers of HD which can be used to predict the onset, monitor progression, and/or evaluate the efficacy of therapeutic treatment are in high demand. The progression of HD is currently evaluated using the Unified Huntington's Disease Rating Scale (UHDRS) in clinics [18, 19]. Nonetheless, the UHDRS tends to be subjective, and its sensitivity to disease progression is low [20]. Tremendous efforts have been devoted to searching for precise and reliable biomarkers using various approaches including neuroimaging and biochemical analyses [21-31]. Considering that hearing tests are generally accessible to HD patients in local clinics, we reasoned that hearing loss may be considered a new feature of HD patients in clinics as well as a potential biomarker for assessing therapeutic interventions for HD.

*Besides the inferior expression of CKB, alterations in mitochondrial functions* were extensively explored in HD. Well-documented mitochondrial abnormalities including dysregulation of a mitochondrial biogenesis co-activator (peroxisome proliferator-activated receptor gamma coactivator-1α, PGC-1α) [32], abnormal calcium homeostasis [33], impaired mitochondrial trafficking [34, 35], and ATP depletion [36] were reported in animals with HD. These findings suggest that energy deficits are critical for the pathogenesis of HD. Oxidation of CKB, which leads to its reduced activity, was also reported in the brain of rodents and humans with HD [37, 38]. Interestingly, decreased levels of CKB in the blood buffy coat fraction were found to be associated with presymptomatic and manifesting HD patients [39], suggesting a potential application of CKB as a biomarker to predict the onset and monitor the progression of HD. It is important to note that downregulation of CKB was also found in numerous neurodegenerative disorders such as Alzheimer's disease, Pick's disease, and diffuse Lewy body disease [40, 41]. Given the importance of CKB in maintaining energy homeostasis and appropriate neuronal functions, it is worth evaluating whether the level of CKB in white blood cells can serve as a reliable biomarker to assess the progression of neurodegenerative diseases (including HD) in which the level of CKB is reduced in the affected brain region(s).

### CKB in the cochlea

Mechanoelectrical transduction of cochlear hair cells in response to acoustic stimuli involves specialized actin-cored microvilli called stereocilia, the deflection of which leads to potassium influx from the endolymph, depolarizes hair cells, and in turn opens voltage-gated calcium channels in cell membranes. The influx of calcium triggers neurotransmitter release from the basal end of the cell into the auditory nerve endings and fires the fiber. This sound reception process in the cochlea requires energy-intense processes to adequately prime the hair bundle movement, for homeostatic calcium regulation, and for potassium recycling to repeat the cycle [42, 43]. Because hair bundles contain no mitochondria, an efficient energy supply mechanism to maintain a sufficient ATP level for immense energy consumption processes in hair bundles [44], such as slow and fast adaptation [45], is crucial. The PCr-CK system thus plays a critical role in managing high-energy demands in the cochlea as demonstrated using a CKB-knockout mouse model that exhibited preferential high-tone hearing loss [44].

Besides cochlear hair cells, CKB is also localized in the inner ear spiral ligament, where several ion transport enzymes such as Na, K-ATPase, and carbonic anhydrase are expressed to facilitate potassium ion cycling back to the endolymph [46-48]. Although the role of CKB in the cochlear lateral wall remains unclear, it is reasonable to propose that the PCr-CK system may also function to shuttle high-energy phosphate to replenish ATP at these intracellular sites where ATPase hydrolyzes ATP to mediate specialized energy demands. It was noted that strial atrophy in aged rodent cochleae is associated with an abnormal expression profile of CKB [46], suggesting an energy-supplying role of CKB during disturbed metabolic demands in strial atrophy.

### ROS-related hearing impairment and the antioxidative role of creatine

Age-related sensorineural hearing loss (SNHL) is the most common sensory deficit in the elderly population [49] and is closely associated with accumulated oxidative damage caused by ROS [50-53]. As discussed above, cochleae possess metabolically active tissues that tend to produce ROS through mitochondrial oxidation. Normally, ROS produced by mitochondria during physiological conditions are scavenged by endogenous antioxidant mechanisms [54-56]. However, when excess ROS following noise overstimulation or ototoxic drug insults overwhelm a cell's natural antioxidant defenses, elevated oxidative stress is known to contribute to several types of hearing impairment, including age-related, noise-induced, and ototoxic drug-induced SNHL [43, 53]. The accumulation of ROS leads to genetic and cellular alterations which cause cellular dysfunctions such as lipid peroxidation, polysaccharide depolymerization, nucleic acid disruption, oxidation of sulfhydryl groups, and enzyme inactivation [57], consequently leading to permanent cochlear degeneration [58-60]. Moreover, a decline in the mitochondrial respiratory function and an increase in the mitochondrial ROS production may render cells more susceptible to apoptosis. Conversely, accumulating evidence demonstrated that antioxidants and free radical scavengers may serve as effective therapeutic agents to block ROS-related activation of death mechanisms in multiple systems, including the auditory system [43, 61-65].

Creatine is a nitrogenous organic acid which is known to increase muscle mass and performance, prevent disease-induced muscle atrophy, and facilitate supplying energy to cells under a reversible catalyzing reaction with CK. Besides its role in energy replenishment, creatine also exerts a strong antioxidant effect by reducing the intra-mitochondrial production of ROS, as well as elevating and preserving the mitochondrial membrane potential [66]. In a noise-induced hearing loss animal model, creatine treatment was shown to significantly attenuate the resultant auditory threshold shifts [67], suggesting that both the maintenance of ATP levels and the scavenging of free radicals mediated by creatine are essential for hearing protection from such oxidative damage. Since oxidative damage caused by ROS has become a common pathological cause involved in several types of hearing loss, creatine supplements are believed to improve the mitochondrial antioxidant defense system and maintain optimal energy homeostasis. Further experiments are needed to further explore the pleiotropic roles of dietary-supplemented creatine in the auditory system.

### Conclusions

Impaired energy homeostasis recently emerged as an important player in a wide variety of neurodegenerative diseases. Our earlier findings of the functional roles of CKB and the PCr-CK system in hearing loss during HD progression strengthen the importance of energy deficits in HD pathogenesis. Potential applications of dietary creatine supplements and approaches that enhance the expression of CKB for degenerative diseases (including HD) with energy deficiency and SNHL warrant further investigations.

## Nonstandard Abbreviations:

CK: creatine kinase
CKB: brain-type creatine kinase
Htt: Huntingtin
HD: Huntington's disease
NII: neuronal intranuclear inclusion
PCr: phosphocreatine
polyQ: polyglutamine
SNHL: sensorineural hearing loss
UHDRS: Unified Huntington's Disease Rating Scale

## References

[R1] Martin JB, Gusella JF (1986). Huntington's disease. Pathogenesis and management. N Engl J Med.

[R2] Orth M, Cooper JM, Bates GP, Schapira AH (2003). Inclusion formation in Huntington's disease R6/2 mouse muscle cultures. J Neurochem.

[R3] Sathasivam K, Hobbs C, Turmaine M, Mangiarini L, Mahal A, Bertaux F, Wanker EE, Doherty P, Davies SW, Bates GP (1999). Formation of polyglutamine inclusions in non-CNS tissue. Hum Mol Genet.

[R4] Tanaka M, Machida Y, Niu S, Ikeda T, Jana NR, Doi H, Kurosawa M, Nekooki M, Nukina N (2004). Trehalose alleviates polyglutamine-mediated pathology in a mouse model of Huntington disease. Nat Med.

[R5] Pattison JS, Robbins J (2008). Protein misfolding and cardiac disease: establishing cause and effect. Autophagy.

[R6] Pattison JS, Sanbe A, Maloyan A, Osinska H, Klevitsky R, Robbins J (2008). Cardiomyocyte expression of a polyglutamine preamyloid oligomer causes heart failure. Circulation.

[R7] Lin YS, Chen CM, Soong BW, Wu YR, Chen HM, Yeh WY, Wu DR, Lin YJ, Poon PW, Cheng ML, Wang CH, Chern Y (2011). Dysregulated brain creatine kinase is associated with hearing impairment in mouse models of Huntington disease. J Clin Invest.

[R8] Wyss M, Kaddurah-Daouk R (2000). Creatine and creatinine metabolism. Physiol Rev.

[R9] Wallimann T, Wyss M, Brdiczka D, Nicolay K, Eppenberger HM (1992). Intracellular compartmentation, structure and function of creatine kinase isoenzymes in tissues with high and fluctuating energy demands: the ‘phosphocreatine circuit’ for cellular energy homeostasis. The Biochemical journal.

[R10] Wallimann T (2007). Introduction–creatine: cheap ergogenic supplement with great potential for health and disease. Sub-cellular biochemistry.

[R11] Payne RM, Haas RC, Strauss AW (1991). Structural characterization and tissue-specific expression of the mRNAs encoding isoenzymes from two rat mitochondrial creatine kinase genes. Biochimica et biophysica acta.

[R12] Jacobus WE, Lehninger AL (1973). Creatine kinase of rat heart mitochondria. Coupling of creatine phosphorylation to electron transport. J Biol Chem.

[R13] Booth RF, Clark JB (1978). Studies on the mitochondrially bound form of rat brain creatine kinase. The Biochemical journal.

[R14] Wallimann T, Walzthony D, Wegmann G, Moser H, Eppenberger HM, Barrantes FJ (1985). Subcellular localization of creatine kinase in Torpedo electrocytes: association with acetylcholine receptor-rich membranes. J Cell Biol.

[R15] Wallimann T, Wegmann G, Moser H, Huber R, Eppenberger HM (1986). High content of creatine kinase in chicken retina: compartmentalized localization of creatine kinase isoenzymes in photoreceptor cells. Proc Natl Acad Sci U S A.

[R16] Wallimann T, Moser H, Zurbriggen B, Wegmann G, Eppenberger HM (1986). Creatine kinase isoenzymes in spermatozoa. J Muscle Res Cell Motil.

[R17] Nuss JE, Amaning JK, Bailey CE, DeFord JH, Dimayuga VL, Rabek JP, Papaconstantinou J (2009). Oxidative modification and aggregation of creatine kinase from aged mouse skeletal muscle. Aging.

[R18] Shoulson I, Fahn S (1979). Huntington disease: clinical care and evaluation. Neurology.

[R19] Huntington Study Group (1996). Unified Huntington's Disease Rating Scale: reliability and consistency. Mov Disord.

[R20] Paulsen JS, Hayden M, Stout JC, Langbehn DR, Aylward E, Ross CA, Guttman M, Nance M, Kieburtz K, Oakes D, Shoulson I, Kayson E, Johnson S, Penziner E (2006). Preparing for preventive clinical trials: the Predict-HD study. Arch Neurol.

[R21] Rosas HD, Feigin AS, Hersch SM (2004). Using advances in neuroimaging to detect, understand, and monitor disease progression in Huntington's disease. NeuroRx.

[R22] Rosas HD, Tuch DS, Hevelone ND, Zaleta AK, Vangel M, Hersch SM, Salat DH (2006). Diffusion tensor imaging in presymptomatic and early Huntington's disease: Selective white matter pathology and its relationship to clinical measures. Mov Disord.

[R23] Henley SM, Bates GP, Tabrizi SJ (2005). Biomarkers for neurodegenerative diseases. Curr Opin Neurol.

[R24] Underwood BR, Broadhurst D, Dunn WB, Ellis DI, Michell AW, Vacher C, Mosedale DE, Kell DB, Barker RA, Grainger DJ, Rubinsztein DC (2006). Huntington disease patients and transgenic mice have similar pro-catabolic serum metabolite profiles. Brain.

[R25] Mochel F, Charles P, Seguin F, Barritault J, Coussieu C, Perin L, Le Bouc Y, Gervais C, Carcelain G, Vassault A, Feingold J, Rabier D, Durr A (2007). Early energy deficit in Huntington disease: identification of a plasma biomarker traceable during disease progression. PLoS One.

[R26] Valenza M, Rigamonti D, Goffredo D, Zuccato C, Fenu S, Jamot L, Strand A, Tarditi A, Woodman B, Racchi M, Mariotti C, Di Donato S, Corsini A, Bates G, Pruss R, Olson JM, Sipione S, Tartari M, Cattaneo E (2005). Dysfunction of the cholesterol biosynthetic pathway in Huntington's disease. J Neurosci.

[R27] Strand AD, Aragaki AK, Shaw D, Bird T, Holton J, Turner C, Tapscott SJ, Tabrizi SJ, Schapira AH, Kooperberg C, Olson JM (2005). Gene expression in Huntington's disease skeletal muscle: a potential biomarker. Hum Mol Genet.

[R28] Runne H, Kuhn A, Wild EJ, Pratyaksha W, Kristiansen M, Isaacs JD, Regulier E, Delorenzi M, Tabrizi SJ, Luthi-Carter R (2007). Analysis of potential transcriptomic biomarkers for Huntington's disease in peripheral blood. Proc Natl Acad Sci U S A.

[R29] Borovecki F, Lovrecic L, Zhou J, Jeong H, Then F, Rosas HD, Hersch SM, Hogarth P, Bouzou B, Jensen RV, Krainc D (2005). Genome-wide expression profiling of human blood reveals biomarkers for Huntington's disease. Proc Natl Acad Sci U S A.

[R30] Zabel C, Chamrad DC, Priller J, Woodman B, Meyer HE, Bates GP, Klose J (2002). Alterations in the mouse and human proteome caused by Huntington's disease. Mol Cell Proteomics.

[R31] Zabel C, Klose J (2004). Influence of Huntington's disease on the human and mouse proteome. Int Rev Neurobiol.

[R32] Cui L, Jeong H, Borovecki F, Parkhurst CN, Tanese N, Krainc D (2006). Transcriptional repression of PGC-1alpha by mutant huntingtin leads to mitochondrial dysfunction and neurodegeneration. Cell.

[R33] Lim D, Fedrizzi L, Tartari M, Zuccato C, Cattaneo E, Brini M, Carafoli E (2008). Calcium homeostasis and mitochondrial dysfunction in striatal neurons of Huntington disease. J Biol Chem.

[R34] Chang DT, Rintoul GL, Pandipati S, Reynolds IJ (2006). Mutant huntingtin aggregates impair mitochondrial movement and trafficking in cortical neurons. Neurobiol Dis.

[R35] Li XJ, Orr AL, Li S (2009). Impaired mitochondrial trafficking in Huntington's disease. Biochim Biophys Acta.

[R36] Milakovic T, Johnson GV (2005). Mitochondrial respiration and ATP production are significantly impaired in striatal cells expressing mutant huntingtin. J Biol Chem.

[R37] Sorolla MA, Reverter-Branchat G, Tamarit J, Ferrer I, Ros J, Cabiscol E (2008). Proteomic and oxidative stress analysis in human brain samples of Huntington disease. Free Radic Biol Med.

[R38] Perluigi M, Poon HF, Maragos W, Pierce WM, Klein JB, Calabrese V, Cini C, De Marco C, Butterfield DA (2005). Proteomic analysis of protein expression and oxidative modification in r6/2 transgenic mice: a model of Huntington disease. Mol Cell Proteomics.

[R39] Kim J, Amante DJ, Moody JP, Edgerly CK, Bordiuk OL, Smith K, Matson SA, Matson WR, Scherzer CR, Rosas HD, Hersch SM, Ferrante RJ (2010). Reduced creatine kinase as a central and peripheral biomarker in Huntington's disease. Biochim Biophys Acta.

[R40] Aksenov MY, Aksenova MV, Payne RM, Smith CD, Markesbery WR, Carney JM (1997). The expression of creatine kinase isoenzymes in neocortex of patients with neurodegenerative disorders: Alzheimer's and Pick's disease. Exp Neurol.

[R41] Aksenova MV, Aksenov MY, Payne RM, Trojanowski JQ, Schmidt ML, Carney JM, Butterfield DA, Markesbery WR (1999). Oxidation of cytosolic proteins and expression of creatine kinase BB in frontal lobe in different neurodegenerative disorders. Dement Geriatr Cogn Disord.

[R42] Spicer SS, Schulte BA (1998). Evidence for a medial K+ recycling pathway from inner hair cells. Hear Res.

[R43] Kopke R, Allen KA, Henderson D, Hoffer M, Frenz D, Van de Water T (1999). A radical demise. Toxins and trauma share common pathways in hair cell death. Ann N Y Acad Sci.

[R44] Shin JB, Streijger F, Beynon A, Peters T, Gadzala L, McMillen D, Bystrom C, Van der Zee CE, Wallimann T, Gillespie PG (2007). Hair bundles are specialized for ATP delivery via creatine kinase. Neuron.

[R45] Gillespie PG, Cyr JL (2004). Myosin-1c, the hair cell's adaptation motor. Annu Rev Physiol.

[R46] Spicer SS, Gratton MA, Schulte BA (1997). Expression patterns of ion transport enzymes in spiral ligament fibrocytes change in relation to strial atrophy in the aged gerbil cochlea. Hear Res.

[R47] Spicer SS, Schulte BA (1992). Creatine kinase in epithelium of the inner ear. J Histochem Cytochem.

[R48] Spicer SS, Schulte BA (1991). Differentiation of inner ear fibrocytes according to their ion transport related activity. Hear Res.

[R49] Someya S, Prolla TA (2010). Mitochondrial oxidative damage and apoptosis in age-related hearing loss. Mech Ageing Dev.

[R50] Head E, Liu J, Hagen TM, Muggenburg BA, Milgram NW, Ames BN, Cotman CW (2002). Oxidative damage increases with age in a canine model of human brain aging. J Neurochem.

[R51] Someya S, Xu J, Kondo K, Ding D, Salvi RJ, Yamasoba T, Rabinovitch PS, Weindruch R, Leeuwenburgh C, Tanokura M, Prolla TA (2009). Age-related hearing loss in C57BL/6J mice is mediated by Bak-dependent mitochondrial apoptosis. Proc Natl Acad Sci U S A.

[R52] Finkel T, Holbrook NJ (2000). Oxidants, oxidative stress and the biology of ageing. Nature.

[R53] Darrat I, Ahmad N, Seidman K, Seidman MD (2007). Auditory research involving antioxidants. Curr Opin Otolaryngol Head Neck Surg.

[R54] Kondratov RV, Vykhovanets O, Kondratova AA, Antoch MP (2009). Antioxidant N-acetyl-L-cysteine ameliorates symptoms of premature aging associated with the deficiency of the circadian protein BMAL1. Aging.

[R55] Roginsky VA, Tashlitsky VN, Skulachev VP (2009). Chain-breaking antioxidant activity of reduced forms of mitochondria-targeted quinones, a novel type of geroprotectors. Aging.

[R56] Massaad CA, Pautler RG, Klann E (2009). Mitochondrial superoxide: a key player in Alzheimer's disease. Aging.

[R57] Southorn PA, Powis G (1988). Free radicals in medicine. I. Chemical nature and biologic reactions. Mayo Clin Proc.

[R58] Seidman MD, Ahmad N, Bai U (2002). Molecular mechanisms of age-related hearing loss. Ageing Res Rev.

[R59] Riva C, Donadieu E, Magnan J, Lavieille JP (2007). Age-related hearing loss in CD/1 mice is associated to ROS formation and HIF target proteins up-regulation in the cochlea. Exp Gerontol.

[R60] Ohinata Y, Miller JM, Altschuler RA, Schacht J (2000). Intense noise induces formation of vasoactive lipid peroxidation products in the cochlea. Brain Res.

[R61] Maekawa H, Matsunobu T, Tsuda H, Onozato K, Masuda Y, Tanabe T, Shiotani A (2009). Therapeutic effect of edaravone on inner ear barotrauma in the guinea pig. Neurochem Int.

[R62] Yamasoba T, Nuttall AL, Harris C, Raphael Y, Miller JM (1998). Role of glutathione in protection against noise-induced hearing loss. Brain Res.

[R63] Seidman MD, Shivapuja BG, Quirk WS (1993). The protective effects of allopurinol and superoxide dismutase on noise-induced cochlear damage. Otolaryngol Head Neck Surg.

[R64] Someya S, Yu W, Hallows WC, Xu J, Vann JM, Leeuwenburgh C, Tanokura M, Denu JM, Prolla TA (2010). Sirt3 mediates reduction of oxidative damage and prevention of age-related hearing loss under caloric restriction. Cell.

[R65] Coleman JK, Kopke RD, Liu J, Ge X, Harper EA, Jones GE, Cater TL, Jackson RL (2007). Pharmacological rescue of noise induced hearing loss using N-acetylcysteine and acetyl-L-carnitine. Hear Res.

[R66] Meyer LE, Machado LB, Santiago AP, da-Silva WS, De Felice FG, Holub O, Oliveira MF, Galina A (2006). Mitochondrial creatine kinase activity prevents reactive oxygen species generation: antioxidant role of mitochondrial kinase-dependent ADP re-cycling activity. J Biol Chem.

[R67] Minami SB, Yamashita D, Ogawa K, Schacht J, Miller JM (2007). Creatine and tempol attenuate noise-induced hearing loss. Brain Res.

